# Two standards of care for HIV: Why are Africans being short-changed?

**DOI:** 10.1186/1742-4690-6-109

**Published:** 2009-12-01

**Authors:** Mark A Wainberg

**Affiliations:** 1 McGill University AIDS Centre, Jewish General Hospital, Montreal, Quebec, Canada H3T 1E2

## 

On World AIDS Day 2009, it is appropriate that we reflect on the accomplishments in HIV therapy that have been made over the past decade. First, we should recognize that the impact of the XIIIth International Conference on AIDS in Durban, South Africa, in 2000 was much more than scientific. It also highlighted the non-acceptability of continuing to deny access to life-saving antiretroviral drugs to HIV-infected people in developing countries. This was in spite of efforts by then President Thabo Mbeki of South Africa to undermine the conference through his ridiculous and irresponsible insinuations that HIV might not be the cause of AIDS. Since 2000, it is estimated that the numbers of people in Africa receiving antiretroviral therapy has increased to approximately four million from about 7000 at the time of the Durban Conference. However, the fact is that almost all HIV-infected persons in developing country settings are today receiving therapies that are considered to be sub-standard by Western criteria.

For example, the triple drug combination that is the most widely prescribed in the world, is termed Triomune. The reason it is so widely used is that it is cheap as well as generically produced, and is therefore extensively used in developing countries where the burden of HIV disease is so extensive. Triomune is a single pill that is taken twice daily. It includes three individual drugs that are termed stavudine (d4T), lamivudine (3TC), and nevirapine (NVP). Of these, it is only 3TC that continues to be widely used by patients in Western countries. Indeed, we have known for at least 7 years that the stavudine component of the Triomune regimen is associated with severe toxicities that can cause facial and body disfiguration [1]. As a result, many patients may ultimately choose to be non-adherent to their antiviral regimens, in part because of the stigmatizing consequences of these deformities. An obvious consequence of this may be unchecked viral replication and the progression of disease.

The problem gets worse. There is now substantial evidence to indicate that patients infected by the subtype C variants of HIV-1 that are predominant in sub-Saharan Africa may be especially prone to develop certain drug resistance-associated mutations in the event that they fail their antiretroviral therapy [2,3]. One of these mutations is the K65R substitution in reverse transcriptase. Unfortunately, this mutation has been shown to be associated with a broad degree of cross-resistance against many members of the nucleoside reverse transcriptase inhibitor (NRTI) family of drugs. As a result, many patients who fail their Triomune regimen will develop the K65R substitution and may be compromised in regard to opportunity to benefit from many second-line therapies that might otherwise check viral replication and HIV disease progression [4]. This is the reason some of the wealthier countries in Africa such as Botswana and Zambia have now switched away from the use of stavudine-based regimens in first-line therapy to instead use other much better tolerated, more efficient drugs such as tenofovir (TDF), that will potentially avoid this problem of cross-resistance.

Fortunately, the World Health Organization has recently recommended that TDF be substituted, wherever possible, for stavudine in the context of initial therapeutic regimens in developing countries. However, it may take years for poorer countries to be able to access alternate medicines such as TDF, which costs at least twice as much as stavudine, even if it is generically produced. In all likelihood as well, generic manufacturers of Triomune, that have stockpiled huge amounts of this antiquated triple cocktail formulation, will be unhappy if their amassed drug supplies do not make their way to market.

Another major issue in regard to the clinical management of HIV-infected individuals in developing countries involves the performance of PCR-based tests to monitor levels of viral RNA in plasma. It has long been argued by some that this test is expensive and that the money involved in performing clinical monitoring of plasma viremia might be better spent on providing access to drugs. However, it is important to understand that the monitoring of plasma viremia represents an essential component of HIV clinical management in all Western countries and provides key information in regard to whether a drug regimen is working or not. Indeed, it is considered to be unacceptable if a patient's viral load in plasma cannot be brought down to and maintained below levels of dectability of the test, i.e. < 50 copies of viral RNA per ml of plasma.

The reason that it is essential to offer plasma viremia testing is simple. Once HIV plasma viremia begins to rise, this is usually a sure indication that the therapeutic regimen being used has probably failed, most likely for reasons of HIV drug resistance as a consequence of drug-selected mutations within the target genes of HIV therapy i.e. reverse transcriptase, protease, and integrase.

It is not only that these mutations can render antiretroviral drugs less useful than they might otherwise be but, in addition, because such mutations can sequentially accumulate if a patient continues to be treated with a drug that is being employed during treatment failure. This, in turn, can lead to higher levels of drug resistance that might otherwise occur as well as to the potential for sexual transmission of resistance-associated mutations.

In certain instances, a more serious consequence may be that the accumulation of drug resistance mutations may prevent newer compounds that represent a next generation of drug development from being effective. For example, the non-nucleoside reverse transcriptase inhibitor (NNRTI) etravirine (ETV) is an excellent compound that maintains efficacy against viruses that contain the most common mutations associated with resistance against two first-generation NNRTIs, NVP and efavarenz (EFV). However, if patients remain on either of these drugs once the most common of these mutations, i.e. K103N, has appeared, they will be likely to develop a wide array of other NNRTI-associated mutations, that will compromise the use of ETV [5].

The problem with monitoring patients by means other than plasma viremia, such as CD4 counts, is that the latter are not well-suited to provide an accurate assessment of the state of viral replication within infected individuals. Although CD4 monitoring is essential and provides vital information in regard to the status of the immune system, it is well known that drops in levels of CD4 counts usually take place long after plasma viremia has started to increase. As a consequence, patients who remain on non-effective triple drug regimens will continue to accumulate drug resistance-associated mutations during periods of relative CD4 stability, since it is only the long-term consequences of unchecked viral replication that lead to CD4 declines. While there is now a trend toward encouraging the use of viral load diagnostics in all developing country settings, this recommendation may have come too late for millions of individuals who have already accumulated multiple drug resistance mutations in their HIV genomes, thereby compromising future therapies.

An additional major difference between therapeutic standards in Western vs developing countries involves the question of when to start therapy. There is now consensus in Western countries that patients ought to begin therapy when their CD4 counts are still above 350 cells/ml^3 ^[6]. Allowing CD4 levels to decline below this level prior to initiation of therapy is associated with considerable risk in regard to both short and long-term responsiveness to highly active antiretroviral therapy (HAART). This is doubtless because of the irreparable damage that is inflicted by HIV replication on the immune system and a relative inability to recover full immune function despite the use of antiretroviral drugs. Unfortunately, most people, even in Western countries, are still unlikely to begin therapy with CD4 counts above 350, the reason being that most individuals are only diagnosed once their CD4 counts have slipped to levels below 250 cells/mm^3^, often because they may then decide to seek medical advice after first developing AIDS-related symptoms.

Efforts are now being made in all Western settings to try to identify HIV-infected individuals far earlier than was previously the case for several reasons. Among these are the potential public health benefits of beginning antiretroviral therapy earlier, such that viral load will quickly diminish and infected individuals will be less likely to transmit virus to others [7]. A second obvious benefit will involve the long term health of infected individuals themselves, if they are able to begin therapy earlier.

In contrast, most individuals in developing countries remain unlikely to be diagnosed with CD4 counts that are above 200. It will take monumental efforts to bring more widespread and efficacious testing for HIV to developing country settings in order that patients may benefit from HAART to the same extent as has occured in the West. This problem is also complicated by the fact that stigmatization of HIV disease is far worse in many developing countries than is the case, for example, in Western Europe and North America [8].

As if the above were not enough, there is even concern that the generic drugs being made available in some developing countries may not be of the same quality as those that are produced by major pharmaceutical companies [9]. It is vital that high standards of bioequivalence be maintained in all countries in which generic HIV drugs are employed, since the use of drugs with fewer active components will increase both the rate of emergence of drug-resistant HIV variants and their transmission. Some observers have even raised the possibility that fake drugs might have found their way to the market in some developing countries.

In short, considerable progress has been made in developing settings, including most African countries, in regard to HIV drug access. However, enormous chasms in regard to standards of care, diagnostic services, and qualities of drugs employed continue to distinguish developing from developed countries, as has been previously pointed out [10]. It is simply not true that HIV disease has been transformed for most people into a chronic manageable condition in a developing country context, in the way that we hold this to be true in the West. Although international bodies such as the World Health Organization are trying to resolve some of these issues, as will be reflected in new sets of guidelines to be published shortly, implementation of change will not be easy or fast. We must all work harder to ensure that these gaps shrink as quickly as possible if we are to realize the ideals and dreams of the XIIIth International Conference on AIDS that was held in Durban only nine years ago.

Mark A. Wainberg is Professor of Medicine and of Microbiology at McGill University in Montreal where he is the Director of the McGill AIDS Centre at the Jewish General Hospital. He was the President of the International AIDS Society between 1998-2000 with responsibility for organization of the XIIIth International Conference on AIDS that was held in Durban, South Africa during July 2000.

## References

[B1] Joly V Flandre P Meiffredy V Leturque N Harel M Aboulker JP Yeni P Increased risk of lipoatrophy under stavudine in HIV-1-infected patients: results of a substudy from a comparative trial AIDS 2002 16 18 2447 54 10.1097/00002030-200212060-00010 12461419

[B2] Doualla-Bell F Avalos A Brenner B Gaolathe T Mine M Gaseitsiwe S Oliveira M Moisi D Ndwapi N Moffat H Essex M Wainberg MA High Prevalence of the K65R Mutation in Human Immunodeficiency Virus Type 1 Subtype C-Infected Batswana Patients Treated with ddI-based regimens Antimicrob Agents Chemother 2006 50 12 4182 5 17015626 10.1128/AAC.00714-06 PMC1693987

[B3] Hosseinipour MC van Oosterhout JJ Weigel R Phiri S Kamwendo D Parkin N Fiscus SA Nelson JA Eron JJ Kumwenda J The public health approach to identify antiretroviral therapy failure: high-level nucleoside reverse transcriptase inhibitor resistance among Malawians failing first-line antiretroviral therapy AIDS 2009 23 9 1127 34 10.1097/QAD.0b013e32832ac34e 19417582 PMC2896488

[B4] Invernizzi CF Coutsinos D Oliveira M Moisi D Brenner BG Wainberg MA Signature nucleotide polymorphisms at positions 64 and 65 in reverse transcriptase favor the selection of the K65R resistance mutation in HIV-1 subtype C J Infect Dis 2009 200 8 1202 6 10.1086/605894 19764886

[B5] Vingerhoets J Peeters M Azijn H Tambuyzer L Hoogstoel A Nijs N de Bethune MP Picchio G An update of the list of NNRTI mutations associated with decreased virological response to etravirine (ETR): multivariate analysis on the pooled DUET-1 and DUET-2 clinical trial data Antivir Ther 2008 13 Suppl 3 A26

[B6] Kitahata MM Gange SJ Abraham AG Merriman B Saag MS Justice AC Hogg RS Deeks SG Eron JJ Brooks JT Rourke SB Gill MJ Bosch RJ Martin JN Klein MB Jacobson LP Rodriguez B Sterling TR Kirk GD Napravnik S Rachlis AR Calzavara LM Horberg MA Silverberg MJ Gebo KA Goedert JJ Benson CA Collier AC Van Rompaey SE Crane HM McKaig RG Lau B Freeman AM Moore RD NA-ACCORD Investigators Effect of early versus deferred antiretroviral therapy for HIV on survival N Engl J Med 2009 360 18 1815 26 10.1056/NEJMoa0807252 19339714 PMC2854555

[B7] Quinn TC Wawer MJ Sewankambo N Serwadda D Li C Wabwire-Mangen F Meehan MO Lutalo T Gray RH Viral load and heterosexual transmission of human immunodeficiency virus type 1. Rakai Project Study Group N Engl J Med 2000 342 13 921 9 10.1056/NEJM200003303421303 10738050

[B8] Wainberg MA HIV transmission should be decriminalized: HIV prevention programs depend on it Retrovirology 2008 5 108 19046412 10.1186/1742-4690-5-108 PMC2613399

[B9] Byakika-Tusiime J Chinn LW Oyugi JH Obua C Bangsberg DR Kroetz DL Steady state bioequivalence of generic and innovator formulations of stavudine, lamivudine, and nevirapine in HIV-infected Ugandan adults PLoS One 2008 3 12 e3981 19096711 10.1371/journal.pone.0003981 PMC2602850

[B10] Jeang KT World AIDS Day 2007: AIDS at 26, are we there yet? Retrovirology 2007 4 86 18053150 10.1186/1742-4690-4-86 PMC2169258

